# Molecular signature of clinical severity in recovering patients with severe acute respiratory syndrome coronavirus (SARS-CoV)

**DOI:** 10.1186/1471-2164-6-132

**Published:** 2005-09-21

**Authors:** Yun-Shien Lee, Chun-Houh Chen, Angel Chao, En-Shih Chen, Min-Li Wei, Lung-Kun Chen, Kuender D Yang, Meng-Chih Lin, Yi-Hsi Wang, Jien-Wei Liu, Hock-Liew Eng, Ping-Cherng Chiang, Ting-Shu Wu, Kuo-Chein Tsao, Chung-Guei Huang, Yin-Jing Tien, Tzu-Hao Wang, Hsing-Shih Wang, Ying-Shiung Lee

**Affiliations:** 1 Genomic Medicine Research Core Laboratory, Chang Gung Memorial Hospital (CGMH), Tao-Yuan, Taiwan; 2 Department of Biotechnology, Ming Chuan University, Tao-Yuan, Taiwan; 3 Institute of Statistical Science, Academia Sinica, Taipei, Taiwan; 4 Department of Obstetrics and Gynecology, Lin-Kou Medical Center, CGMH, Tao-Yuan, Taiwan; 5 Graduate Institute of Clinical Medical Sciences, College of Medicine, Chang Gung University, Tao-Yuan, Taiwan; 6 Department of Pediatrics, Kaohsiung Medical Center, CGMH, Kaohsiung, Taiwan; 7 Department of Internal Medicine, Division of Pulmonary and Critical Care Medicine, Kaohsiung Medical Center, CGMH, Kaohsiung, Taiwan; 8 Department of Internal Medicine, Division of Infectious Diseases, Kaohsiung Medical Center, CGMH, Kaohsiung, Taiwan; 9 Department of Pathology, Kaohsiung Medical Center, CGMH, Kaohsiung, Taiwan; 10 Department of Internal Medicine, Division of Infectious Diseases, Lin-Kou Medical Center, CGMH, Tao-Yuan, Taiwan; 11 Clinical Virology Laboratory, Department of Clinical Pathology, CGMH, Tao-Yuan, Taiwan; 12 Institute of Statistics, National Central University, Tao-Yuan, Taiwan

## Abstract

**Background:**

Severe acute respiratory syndrome (SARS), a recent epidemic human disease, is caused by a novel coronavirus (SARS-CoV). First reported in Asia, SARS quickly spread worldwide through international travelling. As of July 2003, the World Health Organization reported a total of 8,437 people afflicted with SARS with a 9.6% mortality rate. Although immunopathological damages may account for the severity of respiratory distress, little is known about how the genome-wide gene expression of the host changes under the attack of SARS-CoV.

**Results:**

Based on changes in gene expression of peripheral blood, we identified 52 signature genes that accurately discriminated acute SARS patients from non-SARS controls. While a general suppression of gene expression predominated in SARS-infected blood, several genes including those involved in innate immunity, such as defensins and eosinophil-derived neurotoxin, were upregulated. Instead of employing clustering methods, we ranked the severity of recovering SARS patients by generalized associate plots (GAP) according to the expression profiles of 52 signature genes. Through this method, we discovered a smooth transition pattern of severity from normal controls to acute SARS patients. The rank of SARS severity was significantly correlated with the recovery period (in days) and with the clinical pulmonary infection score.

**Conclusion:**

The use of the GAP approach has proved useful in analyzing the complexity and continuity of biological systems. The severity rank derived from the global expression profile of significantly regulated genes in patients may be useful for further elucidating the pathophysiology of their disease.

## Background

SARS-CoV is a single-stranded, plus-sense RNA virus with a genome of ~30 kb. Its sequence does not closely resemble any of the previously characterized coronaviruses [1-4]. Before SARS-CoV was recognized as the cause of the deadly SARS [1-3,5-7], other human coronaviruses had only been known to account for 15–30% of colds [8]. SARS-CoV appears to be new to humans, as supported by the finding that human sera collected before the SARS outbreak did not contain antibodies against this virus [3,9]. After an incubation period from 2 to 10 days, SARS patients might develop fever (>38°C), headache, dry cough, and pneumonia [3,5,9-14]. Most patients gradually recovered while some progressed to respiratory distress syndrome with ~10% mortality rate. The genome-wide changes in human gene expression when challenged by this novel pathogen are essentially unknown.

Profiles of gene expression patterns help define the complex biological processes associated with both health and disease *in vivo*. Investigation of host responses to infection with *in vitro *models have offered insights into mechanisms of pathogenesis, and have highlighted the potential for applications of microarray technology to diagnose infection *in vivo *[15]. Whitney et al. observed that the variation in gene expression patterns in the blood of healthy subjects was strikingly smaller than the significant changes induced by diseases either in patients with cancer or with bacterial infections [15]. It was conceivable that microarray profiling of gene expression in whole bloods exhibits the potential in monitoring the patients' responses to a disease, especially a novel infection such as SARS.

Many discriminative methods have been developed for analysis of microarray gene expression data in cancer patients and the resulting classifications have been correlated closely with clinical parameters [16-19]. For instance, the discovery of signature genes for breast cancers through microarray analysis of gene expression has provided us with a more precise clinical staging that will improve the outcome of treatment [20,21]. However, clinical parameters are not always in a discrete pattern but more likely in a continuous fashion, where an absolute classification may not be achievable. Herein we present the use of cDNA microarray analysis of gene expression in whole blood from a cohort of recovering SARS patients, of whom the disease severity appeared to be a continuum. After we had identified the molecular signature of 52 genes that accurately discriminated acute SARS patients from non-SARS controls, we ranked the disease severity of these patients using a generalized association plot (GAP) elliptical seriation algorithm [22] based on the expression profiles of the 52 genes. The derived severity rank of the patients proved to be closely correlated with their clinical parameters, namely, the recovery period (in days) and the clinical pulmonary infection score.

## Results

### Patient information

Using the cDNA microarrays spotted with duplicated 7,334 cDNA clone, we analyzed RNA specimens successfully amplified in 44 peripheral blood collected from 25 confirmed SARS patients (age ranged from 23 to 80 years old, mean = 41.8, SD = 17.2, median = 34), of whom 24 survived. Except for one patient who died on the 4th day, duration of hospitalization in this cohort ranged from 12 to 51 days (n = 24, mean = 24.5, SD = 10.1, median = 21) (Additional file 1). We defined 11 specimens as acute SARS (AS) using the following criteria: (i) the whole blood RNA from a hospitalized patient was PCR positive for SARS-CoV, or (ii) the specimen was collected within 10 days after the disease onset in patients whose blood was later diagnosed ELISA-positive for anti-SARS IgG. The rest of 33 RNA specimens from SARS patients were labelled as recovering SARS (RS). Our study included 11 normal control (NC) volunteers and 11 patients with bacterial infections (IN) as healthy and non-SARS infection controls, respectively (Additional files 2 and 3).

### cDNA microarray analysis

**Figure 1:**
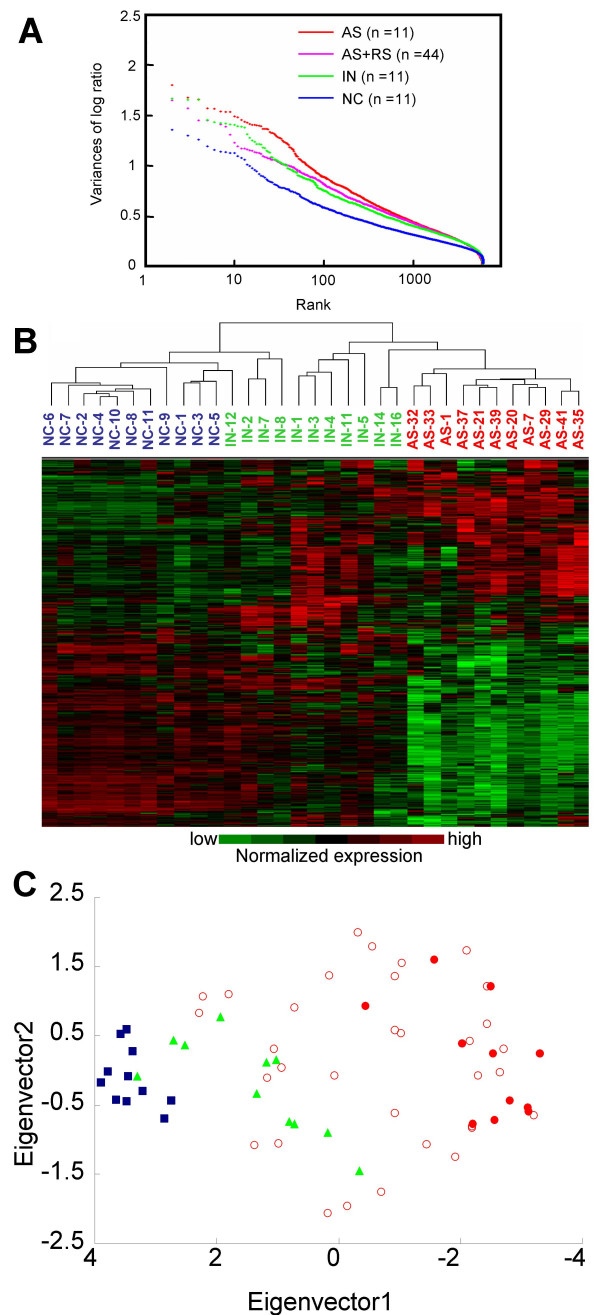
**Significant differences in gene expression profiles in patients with SARS or bacterial infection**. Using a probe set with 6,525 annotated genes, global gene expression was analyzed by **(A) **variation distribution in peripheral blood specimens from patients with acute SARS (AS), recovering SARS (RS), bacterial infections (IN), and normal controls (NC). **(B) **In the hierarchical clustering of relative change in gene expression using a probe set of 885 filtered genes (gene vector >0.5 SD), red indicates upregulation and green indicates downregulation in gene expression relative to a common reference that was the pooled amplified RNA from 11 normal controls. **(C) **Using the 885-gene set, singular value decomposition (SVD) analysis by two eigenvectors showed three distinguished clusters of AS (red ●), IN (green ▲), and NC (blue ▮) groups, with the RS (red ○) specimens scattering among AS and IN.

When we compared the gene expression profiles among acute SARS (AS), recovering SARS (RS), bacterial infection (IN), and normal control (NC) groups, we observed the variances of gene expression in both SARS (AS, AS+RS) and bacterial (IN) groups to be equally higher than that in healthy controls (NC) (Fig. 1a). This result indicates that gene expression profiles of either SARS or bacterial groups differed significantly from that of normal controls.

A probe set of 885 genes with standard deviations greater than 0.5 across 66 arrays was selected for further analyses. An average linkage hierarchical clustering tree with Pearson correlation proximity was built on the 33 arrays (11 NC, 11 IN, and 11AS) using these 885 genes (Fig. 1b). The AS and NC groups were well separated into two opposite coherent clusters. Singular value decomposition (SVD) analysis, a dimension reduction method to project gene expression profiles to fewer representative eigenvectors [23], also successfully separated AS, IN, and NC specimens into three clusters with first two eigenvectors (Fig. 1c). Interestingly, the recovering SARS (RS) samples are interspersed among the AS and IN samples.

**Figure 2:**
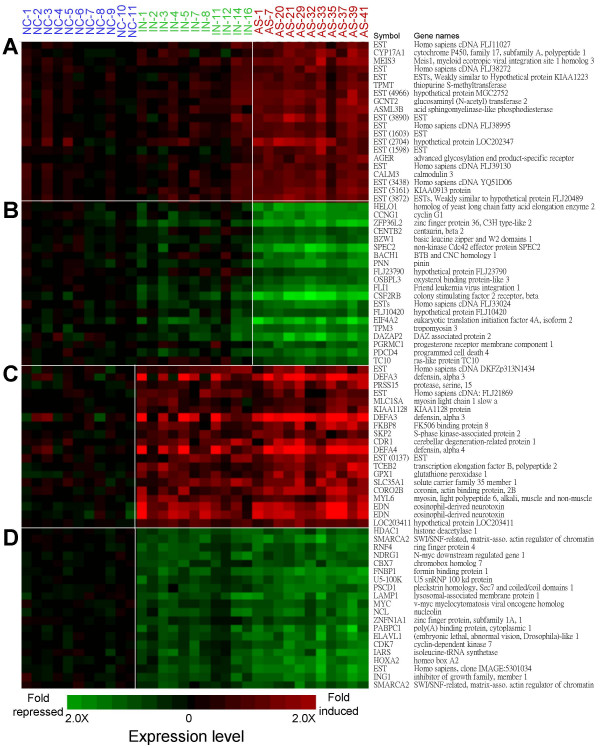
**The top 40 discriminating genes with the highest distinction values for AS or NC groups**. Twenty genes that were specifically **(A) **upregulated or **(B) **downregulated in patients with SARS. Another twenty genes that were non-specifically **(C) **upregulated or **(D) **downregulated by both bacterial infection and SARS. Each column represents an individual sample and each row represents a gene. The color range reflected relative change according to the scale shown. NC, normal control; IN, bacterial infection; AS, acute SARS. GMRCL clone numbers of some ESTs are also included in the parentheses.

To identify which genes were specifically regulated by SARS-CoV, we performed two sets of two-sample Student t-test for means with an unequal-variance assumption. In the first set, we contrasted 11 AS versus 22 non-AS (NC and IN) specimens on all 885 genes. The genes with significant testing results were considered to be specifically induced by SARS-CoV (Fig. 2a,b). For the second set of t-tests, we compared 11 NC with 22 non-NC (IN and AS) specimens. We considered that the change in significant genes identified by the second t-test was induced by both bacterial and viral infections (Fig. 2c,d). Genes identified from these two sets of test were then ranked separately according to the corresponding sets of P-values. Gene expression profiles for the top 20 and the bottom 20 genes from both sets are displayed as Figure 2.

Unexpectedly, most of the genes specifically upreguated by SARS-CoV are ESTs (13/20 genes) that were not annotated previously (Fig. 2a). On the other hand, SARS-CoV stimulated the host innate immunity by upregulating genes including defensins [24,25] and eosinophil derived neurotoxin [26,27], similar to that of bacterial infections (Fig. 2c).

### Signature genes and GAP algorithms

**Figure 3:**
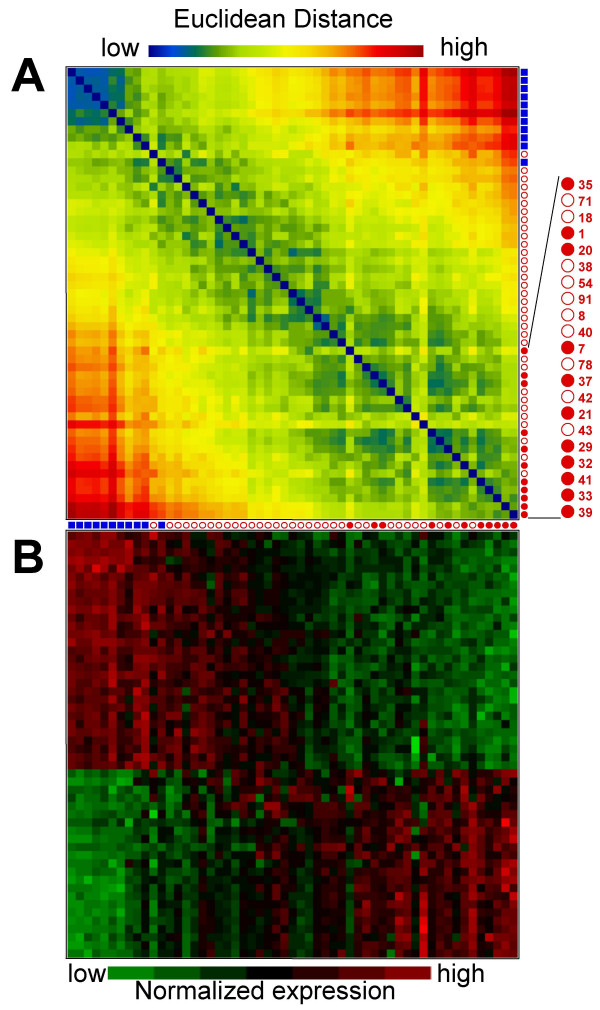
**Generalized associated plots (GAP) analysis of SARS patients samples**. **(A) **Pair-wise Euclidean distance matrix that was sorted by a GAP using 52 genes with the highest discriminating power for AS groups revealed the minimum anti-Robinson events in the matrix, resulting in a smooth transition order of the AS and RS specimens from severely diseased to healthy states. AS (red ●); RS (red ○); NC (blue ▮). **(B) **Gene expression profile for the 52 discriminating genes displayed in the order obtained from the GAP method.

A simple k-nearest-neighbour method was used to obtain a near optimal number of 30 genes from the 885 filtered gene set for discriminating specimens between acute SARS (AS) and non-SARS (NC and IN) (Additional file 4). The selected top 30 upregulated (P < 6 × 10^-6^) and the top 30 downregulated genes (P < 4 × 10^-7^) from the AS versus non-AS (IN and NC) Student's t-test were used as the specific probe set to assess the status of SARS infection. Eight genes that were also significant in the NC versus non-NC (AS and IN) t-test were excluded, resulting in a specific AS probe set of 52 genes. For the GAP analysis, we calculated pair-wise Euclidean distances among 55 samples (11 AS, 33 RS, 11 NC) using these 52 genes, aiming to identify a one-dimensional order that could reflect the severity structure of the disease (Fig. 3a). Using this GAP elliptical arrangement of 55 specimens (columns), we observed a transition of gene expression patterns of 52 genes (rows) from the left side where NC clustered to the right side where AS accumulated (Fig. 3b). Hierarchical clustering trees guided by self-organized map (SOM) and other clustering methods were also performed to sort the SARS patient samples using the same 52 genes. The Robinson criterion [22,28,29] is often employed to assess the performances of different seriation algorithms. Table 1 (and Additional file 5) shows that the GAP algorithm derived a smoother transition pattern than other methods in the Robinson sense. Thereby, we have derived the SARS severity rank according to the expression profile of 52 signature genes as a whole in each patient, as demonstrated by the smooth transition of expression levels in each (row) of these genes from NC to RS to AS (Fig. 3b).

**Table 1:** Performance of Robinson structure with different seriation algorithms.

	Anti-Robinson Events	
Seriation Algorithm	Counts	Scaled Counts (%)

Random Order (NC-AS-RS)	26,245	100.00
Original Order	23,030	87.75
Self Organizing Map (SOM) Order	11,499	43.81
Average Linkage Tree/Original	10,268	39.12
Average Linkage Tree/SOM	10,738	40.91
Average Linkage Tree/GAP	5,940	22.63
GAP Elliptical Order	5,022	19.14

For validation purposes, we further tested the stability of the rank (order) derived from GAP analysis on the 52 genes for the 55 specimens. The same GAP procedure was repeatedly applied to the top 20 to 200 genes (among the filtered 885 genes) with significant p-values (Student's t-test) between the AS versus non-AS (IN and NC). While the ranks for the 55 specimens obtained from the most significant 20 to 200 genes are highly correlated to each other, they are significantly different from the ranks derived from the 52-gene sets that were randomly selected from the 885 genes (data not shown).

We scrutinized the clinical courses of patients who donated the 10 RS specimens that were scattered among AS (Fig. 3a) and found evidence of underlying severity of the disease in the majority of patients. For example, sample RS43 from a patient who had been discharged from hospital for 2 weeks was still PCR-positive for SARS-CoV; RS54, a PCR-positive sample was not grouped as AS because of the negative ELISA result. RS38, RS40, and RS42 still represented acute SARS infections because they were collected only 1, 2, and 3 days after AS37, AS39, and AS41, respectively. Patients with RS78 and RS91 who had severe SARS courses were hospitalized for 41 and 51 days, respectively. The patient for RS8 was in the second week of disease. The only two unexplained specimens, RS18 and RS71 from the same patient, may represent a unique biological variability, accounting for the misclassification using this 52-gene molecular signature.

### Molecular signature for severity and clinical correlations

**Figure 4:**
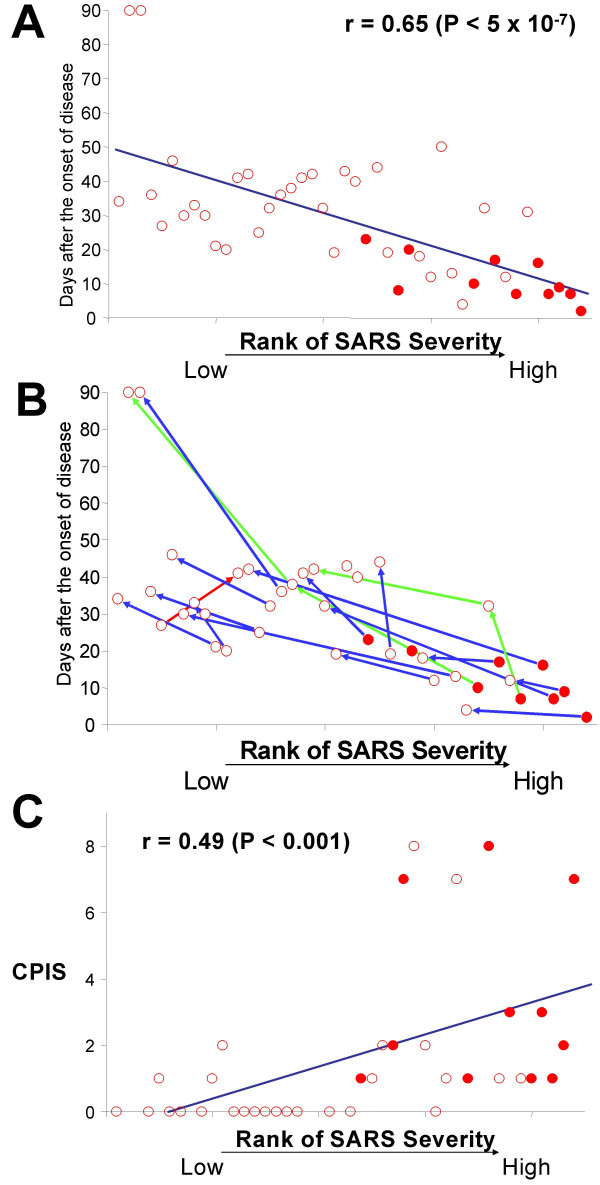
**Correlations between the GAP-derived rank for SARS severity and clinical parameters**. **(A) **The scatter plot of all SARS specimens with the order obtained from the GAP method and the days after the onset of disease showed a significant correlation (P < 5 × 10^-7^). **(B) **Sixteen out of 17 SARS patients who submitted multiple blood specimens showed a similar trend of changes in the GAP-derived severity rank along with the recovery from the disease. Patients with 2 (n = 15) and 3 specimens (n = 2) were labeled with blue and green lines, respectively. **(C) **The scatter plot of all AS and RS specimens with the order obtained from the GAP method and clinical pulmonary infection score (CPIS) showed a significant correlation (P < 0.001).

To test the efficacy of using these 52 genes as the molecular signature for the severity of SARS patients, we identified a significant correlation (P < 1 × 10^-6^) between the derived rank of SARS severity and the number of days after the onset of disease (Fig. 4a). We further used this rank of SARS severity to examine the recovery trend in 17 recovering patients who had donated multiple specimens (Fig. 4b). Except for the one patient (5.3 % = 1/19, shown as the red line in Fig. 4b), similar trends existed in 18 out of 19 lines (94.7 %). Pugin et al. combined body temperature, white blood cell count, volume and appearance of tracheal secretions, oxygenation, chest X-ray, and tracheal aspirate cultures into a clinical pulmonary infection score (CPIS) as a diagnostic tool for pneumonia [30]. We observed that the rank of SARS severity was also significantly (P < 0.001) correlated with the CPIS (Fig. 4c). Collectively, these results demonstrate a correlation between the molecular severity rank and clinical factors, suggesting the usefulness of the molecular signature as a genome-wide parameter for gauging the severity of SARS patients.

## Discussion

Diverse infections can induce a shared core gene expression involving the human innate immune system; each infection may also trigger a pathogen-specific immune response of the host. The innate immune genes were upregulated in both acute SARS (AS) and bacteria infection (IN) patients (Fig. 2c). SARS was a novel viral infection that had not been encountered by the humans in the history before 2003. Intriguingly, most of the genes specifically upreguated in SARS patients were ESTs (13/20 genes) (Fig. 2a), suggesting that the first human encounter with SARS-CoV might provoke a set of human genes that were poorly annotated due to disuse. Annotation of these ESTs may lead to the discovery of novel genes.

Given the high cost of microarray analyses, the detection of a comprehensive gene expression profile may not be cost-effective for clinical diagnosis and evaluation of patients with infectious diseases. However, in a complex system such as the human body where genes interplay through intricate circuitries, it is inadequate to examine only a few routine parameters in biochemistry and blood cell counts for the global physiochemical status of a patient at the time of blood collection. In this report, we applied the GAP method to derive a smooth transition pattern among samples based on the molecular signature consisting of 52 genes, which in turn were used to monitor the severity of clinical courses of SARS patients. Instead of clustering samples into discrete groups in a method similar to commonly-used microarray classifications [31], GAP focuses more on a global orientation of the sample-to-sample relationship. For instance, the AS and RS samples were seriation ranked (Fig. 3), and the rank order proved to correlate well with clinical parameters (Fig. 4).

The GAP-derived rank of severity also provided us with a unique way, where expression of most relevant genes were all considered, to decipher the meaning of the changes in other genes obtained from the same microarray experiment. For instance, we have identified the correlative change in matrix metalloproteinase MMP-7 and MMP-9 (Additional file 6): both can stimulate α-defensin [32]. Importantly, these correlations could not be revealed with other parameters alone, such as number of days after disease onset or clinical score CPIS (data not shown).

In this study, however, there might be technical limitations during RNA isolation from some clinical specimens as well as an unavoidable sample-collecting bias. First, both RNA isolation from SARS specimens and RNA amplification were performed in the Biosafety Level 3 laboratory, where the instrument for RNA quantitation was not available. This limitation resulted in the failed generation of aRNA from 10 out of 54 SARS specimens (Additional file 1). Unfortunately, these 10 specimens contained 7 specimens from patients at an early (i.e. first 2 weeks) stage [14]. Secondly, 25 SARS patients who donated blood specimens for this study may belong to the milder subgroup of a total of 44 SARS patients in Kaohsiung Medical Center of Chang Gung Memorial Hospital. According to a paper describing the complete cohort of SARS patients [33], intubation and mechanical ventilation were required in 20 out of these 44 patients. However, only two in our 25 patients needed intubation (Additional file 1). The aforementioned two potential limitations may account for why our microarray results could not detect a correlation with a possible worsening clinical course before recovering, which was described by Peiris et al [14].

In conclusion, we propose the use of a molecular signature reflecting the severity of SARS in order to interpret the trends of expression changes in groups of genes within particular functional categories. The use of GAP methodology proved to be instrumental in determining the severity of SARS. The derived severity ranking of SARS patients in turn formed a gradual basis for the analysis of the interaction patterns, providing us with a useful tool for understanding the molecular pathogenesis of this novel viral infection.

## Conclusion

We illuminate the human gene expression profiles, in terms of gene expression in peripheral blood, to the unprecedented infection of SARS-CoV. We also discovered a smooth transition pattern of severity from normal controls to acute SARS patients based on the gene expression profiles by generalized associate plots (GAP). The rank of SARS severity was significantly correlated with other clinical parameters.

## Methods

### Patient information and specimen preparation

Blood specimens of 25 SARS patients (Additional file 1) were collected from 10 May to 4 July 2003 at Kaohsiung Medical Center of Chang Gung Memorial Hospital (CGMH) in Kaohsiung City of southern Taiwan. Two additional blood samples (RS94 and RS97) were collected from apparently healthy individuals who had recovered from SARS infection 3 months later. Diagnosis of SARS was based on the guidelines of World Health Organization (WHO) [34]. More comprehensive data of the SARS cohort were previously published [33]. This study was approved by the Institute Review Board of CGMH. Total RNA was isolated with the PAXgene Blood RNA System (Qiagen, USA) and stored at -80°C. After RNAs were further purified and concentrated into 15 μl BR5 solution with RNeasy MinElute kit (Qiagen, USA), 2 μl were used for linear RNA amplification using RiboAmp RNA Amplification Kit (Arcturus, California USA). Before the first Strand Nuclease Mix was added to the RNA samples, all of the RNA purification and amplification were performed inside a Biosafety Level 3 laboratory located in Lin-Kou Medical Center of CGMH. We analyzed the quality and quantity of amplified RNA with Bioanalyzer 2100 (Agilent, CA, USA).

### Anti-SARS-CoV IgG ELISA and real-time quantitative PCR analysis

The antigen used for the SARS detection ELISA was the detergent-extracted and gamma irradiated Vero E6 cells infected with SARS-CoV. Identical preparations from uninfected Vero E6 cells were used as the control. Patients' sera were 1:10 diluted and added to the ELISA plates, and goat anti-human IgG antibody conjugated with horseradish peroxidase (DAKO, Cambridgeshire, UK) was added for enzymatic reaction. After adding the substrate, O-phenylenediamine, the optical density (O.D.) was measured at 450 nm wavelength. The cutoff value of O.D. for SARS-CoV IgG ELISA was 0.15. Sensitivity of this method was 100% (28/28 in confirmed SARS cases) and specificity was 98.4% (790/803) in the healthy control group.

Real-time quantitative PCR analysis for SARS-CoV was performed with Cor-p-F4, Cor-p-R4 and Cor-probe developed by CDC (GA, USA) with HT 7900 Sequence Detection System (Applied Biosystems, CA, USA).

### Microarray procedures

In this study, we used the *GMRCL Human 7K set*, *Version 2 *chips as previously described [35]. Twelve amplified RNA samples from healthy donors (Additional file 3) were pooled as the common reference for every array in this study. A total of 66 aRNA samples including 11 acute SARS (AS), 33 recovering SARS (RS), 11 non-SARS infection (IN), and 11 normal controls (NC) were analyzed with cDNA microarrays as tests against the pooled aRNA (the common reference). Among 66 aRNA preparations, 28 were analyzed with the dye-swapping microarray design. We averaged the log ratios of the duplicated spots on each slide. In the dye swapping experiments, we further averaged the log ratios derived from two slides. We used 400 ng of aRNA for labeling and hybridization using a 3DNA Array 350RP Detection kit (Genisphere, PA, USA), and scanned slides with a confocal scanner ChipReader (Virtek, Canada). We acquired the spot and background intensities with GenePix Pro 4.1 software (Axon Instruments, Inc., CA, USA), and carried out within-slide normalization using programs written with MATLAB 6.0 software (The MathWorks, Inc., MA, USA). To assure the reproducibility of our microarray system, we got the similar gene expression profiles from replicated samples (RS88) using the hierarchical clustering analysis and also got the highly correlated results (r^2 ^= 0.84) from two specimens (AS37 and RS38) that were collected from the same patient at a time interval of only one day. We consistently obtained identical results in each of 28 pairs dye-swapping experiments. The complete microarray data is available in Additional file 7.

### Hierarchical clustering and singular value decomposition

We performed hierarchical clustering using Cluster and TreeView software [36] with the following parameters: (i) a standard deviation > 0.5 as the filtering cutoff point (885 genes with marked changes selected among 66 arrays), (ii) mean-centered genes and normalized genes, (iii) cluster analysis carried out with uncentered correlation of arrays. We also performed a singular value decomposition (SVD) [23] analysis of the correlation matrix for all 66 samples. The first two eigenvectors weighted by the corresponding singular values (eigenvalues) of the 66 samples were plotted against each other.

### Euclidean distance matrix by generalized association plots

Robinson criterion [22,28] is frequently used to assess the performances of sorting algorithms on symmetric proximity matrices. A Robinson Matrix, R = [*r*_*ij*_], is a symmetric matrix such that *r*_*ij *_≤ *r*_*ik *_if *j*<*k*<*i *and *r*_*ij *_≥ *r*_*ik *_if *i*<*j*<*k*. The GAP elliptical seriation [22] utilizing the ellipse structure from a singular value decomposition of a converged correlation coefficient matrix usually identifies permuted matrix with a near Robinson form. A brief review on GAP and some details of its applications are available [37].

## Authors' contributions

Lee YS, Chen CH and Wang TH designed the study and prepared the manuscript. Lee YS, Tien-YJ and Chen CH carried out the statistical analysis. Lee YS, Wang TH, Chen ES, Wei ML, Chen LK carried out the microarray experiments. Chao A, Yang KD, Lin MC, Wang YH, Liu JW, Eng HW, Chiang PC, Wu TS, Tsao KC, Huang CG, Wang HS and Lee YS obtained the clinical materials and analyzed clinical information. All authors read and approved the final manuscript.

## Supplementary Material

Additional File 1 Demographics of SARS Patients. Click here for file

Additional File 2 Demographics of patients with non-SARS infection. Click here for file

Additional File 3 Demographics of healthy donor (information of the pooled reference). Click here for file

Additional File 4 K-nearest-neighbour methods in evaluating the best discriminating (classifying) accuracy for AS and non-SARS specimens. Click here for file

Additional File 5 Euclidean distance matrix of 55 specimens with 52 selected genes using different seriation algorithms. Click here for file

Additional File 6 Analyses of gene expression in MMP-7 and MMP-9, both of which are involved in innate immunity. Click here for file

Additional File 7 Complete microarray data Click here for file
