# Supplementary material for: Molecular signature of clinical severity in recovering patients with severe acute respiratory syndrome coronavirus (SARS-CoV)
Source: BMC Genomics. 2005 Sep 21;6:132. doi: 10.1186/1471-2164-6-132 (PMC1262710; doi:10.1186/1471-2164-6-132)
Supplement: Additional File 1 — Demographics of SARS Patients. [file 1471-2164-6-132-S1.doc]

### Additional file 1.

### Demographics of SARS patients

| **RNA number** | **Patient No.** | **Sex** | **Age**  **(year)** | **Positive SARS status by** | **aRNA** | **Total duration of hospitalization (days)** | **Blood collected on the day**  **after the onset of disease** |
| --- | --- | --- | --- | --- | --- | --- | --- |
| AS-21 | 1 | Male | 80 | PCR, ELISA | Y | 41 | 7 |
| RS-78 |  |  |  |  | Y |  | 32 |
| RS-87 |  |  |  |  | Y |  | 42 |
| AS-32 | 2 | Male | 46 | PCR, ELISA | Y | 21 | 7 |
| RS-69 |  |  |  |  | Y |  | 32 |
| AS-37 | 3 | Female | 50 | PCR | Y | 18 | 17 |
| RS-38 |  |  |  |  | Y |  | 18 |
| AS-41 | 4 | Male | 70 | PCR | Y | 12 | 9 |
| RS-42 |  |  |  |  | Y |  | 12 |
| AS-35 | 5 | Male | 26 | PCR, ELISA | Y | 24 | 23 |
| RS-45 |  |  |  |  | Y |  | 41 |
| AS-1 | 6 | Male | 23 | ELISA | Y | 21 | 8 |
| AS-7 | 7 | Female | 34 | ELISA | Y | 26 | 10 |
| RS-73 |  |  |  |  | Y |  | 38 |
| RS-97 |  |  |  |  | Y |  | > 90 |
| AS-29 | 8 | Female | 27 | PCR | Y | 22 | 16 |
| RS-70 |  |  |  |  | Y |  | 42 |
| AS-39 | 9 * | Male | 60 | PCR | Y | 4 (expired) | 2 |
| RS-40 |  |  |  |  | Y |  | 4 |
| RS-18 | 10 | Female | 51 | ELISA | Y | 21 | 19 |
| RS-71 |  |  |  |  | Y |  | 44 |
| RS-8 | 11 | Female | 27 | ELISA | Y | 13 | 13 |
| RS-50 |  |  |  |  | Y |  | 30 |
| RS-52 | 12 | Male | 24 | ELISA | Y | 20 | 20 |
| RS-82 |  |  |  |  | Y |  | 33 |
| 24 | 13 | Female | 33 | ELISA | N | 21 | 3 |
| RS-53 |  |  |  |  | Y |  | 21 |
| RS-79 |  |  |  |  | Y |  | 34 |
| RS-54 | 14 | Male | 55 | PCR | Y | 18 | 12 |
| RS-61 |  |  |  | (ELISA negative) | Y |  | 19 |
| 23 | 15 | Female | 53 | ELISA | N | 25 | 5 |
| RS-75 |  |  |  |  | Y |  | 25 |
| RS-83 |  |  |  |  | Y |  | 36 |
| 2 | 16 | Female | 45 | ELISA | N | 27 | 6 |
| RS-58 |  |  |  |  | Y |  | 27 |
| RS-86 |  |  |  |  | Y |  | 41 |
| 30 | 17 | Female | 33 | ELISA | N | 19 | 11 |
| RS-57@ |  |  |  | PCR | Y |  | 32 |
| RS-88 |  |  |  |  | Y |  | 46 |
| 16 | 18 * | Female | 53 | ELISA | N | 51 | 12 |
| RS-91 |  |  |  |  | Y |  | 50 |
| 28 | 19 | Male | 25 | ELISA | N | 32 | 18 |
| RS-64 |  |  |  |  | Y |  | 43 |
| 27 | 20 | Female | 24 | ELISA | N | 15 | 13 |
| RS-46 |  |  |  |  | Y |  | 30 |
| 31 | 21 | Female | 26 | ELISA | N | 22 | 11 |
| RS-67 |  |  |  |  | Y |  | 36 |
| RS-94 |  |  |  |  | Y |  | > 90 |
| 9 | 22 | Female | 27 | ELISA | N | 32 | 16 |
| 55 |  |  |  |  | N |  | 33 |
| RS-62 |  |  |  | PCR | Y |  | 40 |
| AS-20 | 23 | Male | 51 | PCR | Y | 49 | 7 |
| AS-33 | 24 | Female | 74 | PCR | Y | 21 | 7 |
| RS-43# | 25 | Female | 28 | PCR, ELISA | Y | 17 | 31 |
| Total | N = 25 | Male =10, Female =15 | **Average = 41.8** |  | N=10  Y = 44 | **Average =**  **24.5 (N = 24)** |  |

# About 2 weeks after being discharged from hospital, patient’s blood was still PCR-positive for SARS-CoV.

* Intubation and mechanical ventilation were required.
