# Supplementary material for: Molecular signature of clinical severity in recovering patients with severe acute respiratory syndrome coronavirus (SARS-CoV)
Source: BMC Genomics. 2005 Sep 21;6:132. doi: 10.1186/1471-2164-6-132 (PMC1262710; doi:10.1186/1471-2164-6-132)
Supplement: Additional File 2 — Demographics of patients with non-SARS infection. [file 1471-2164-6-132-S2.doc]

### Additional file 2.

### Demographics of patients with non-SARS infection.

| **RNA number** | **SEX** | **AGE (year-old)** | **Clinical Diagnosis** | Culture |
| --- | --- | --- | --- | --- |
| IN-1 | Female | 45 | Abdominal abscess | *E. coli,*  *Pseudomonas,* |
| IN-2 | Male | 39 | Urinary tract infection | *Staphylococcus* |
| IN-3 | Female | 70 | Pneumonia | *Staphylococcus* |
| IN-4 | Female | 84 | Tuberculosis | *M. tuberculosis* |
| IN-5 | Male | 72 | Endocarditis | *Streptococcus* |
| IN-7 | Female | 78 | Sepsis | *ND* |
| IN-8 | Female | 56 | Cellulitis | *Staphylococcus* |
| IN-11 | Male | 49 | Sepsis | *ND* |
| IN-12 | Female | 64 | Pneumonia | *Streptococcus,*  *Hemophilus* |
| IN-14 | Male | 45 | Abdominal abscess | *Staphylococcus* |
| IN-16 | Male | 19 | Meningitis | *ND* |

*ND*: not detectable because of the previously empiric use of antibiotics.
