# Supplementary material for: Molecular signature of clinical severity in recovering patients with severe acute respiratory syndrome coronavirus (SARS-CoV)
Source: BMC Genomics. 2005 Sep 21;6:132. doi: 10.1186/1471-2164-6-132 (PMC1262710; doi:10.1186/1471-2164-6-132)
Supplement: Additional File 3 — Demographics of healthy donor (information of the pooled reference). [file 1471-2164-6-132-S3.doc]

**Additional file 3.**

**Demographics of healthy donors (information of the pooled reference).**

| **RNA number** | **Sex** | **Age** | **Microarray results in this study** | **aRNA used in the pooled reference** |
| --- | --- | --- | --- | --- |
| NC-1 | Female | 32 | Y | N |
| NC-2 | Male | 28 | Y | Y |
| NC-3 | Male | 28 | Y | N |
| NC-4 | Female | 27 | Y | Y |
| NC-5 | Female | 51 | Y | Y |
| NC-6 | Male | 46 | Y | N |
| NC-7 | Male | 46 | Y | Y |
| NC-8 | Female | 51 | Y | Y |
| NC-9 | Female | 26 | Y | Y |
| NC-10 | Male | 26 | Y | Y |
| NC-11 | Female | 26 | Y | Y |
| M4 | Male | 28 | N | Y |
| Mb3 | Male | 23 | N | Y |
| F6 | Female | 31 | N | Y |
| F8 | Female | 56 | N | Y |
| Total | Male = 7,  Female = 8 | Average = 35 | 11 | 12 |
