# Supplementary material for: Molecular signature of clinical severity in recovering patients with severe acute respiratory syndrome coronavirus (SARS-CoV)
Source: BMC Genomics. 2005 Sep 21;6:132. doi: 10.1186/1471-2164-6-132 (PMC1262710; doi:10.1186/1471-2164-6-132)
Supplement: Additional File 4 — K-nearest-neighbour methods in evaluating the best discriminating (classifying) accuracy for AS and non-SARS specimens. [file 1471-2164-6-132-S4.doc]

**Additional file 4.**

**K-nearest-neighbour methods in evaluating the best discriminating (classifying) accuracy for AS and non-SARS specimens.**

To obtain an optimal number of genes from the 885 filtered gene set for discriminating specimens between acute SARS (AS) and non-SARS (NC and IN), we set up a simple k-nearest-neighbour classifier. To obtain a symmetric set of genes for discriminating between AS and non-AS specimens, n (1 ≤ n ≤ 100) genes from each of the AS-upregulated and AS-downregulated groups were combined into a 2n gene set. For each n, we randomly sampled n genes from each set and used these 2n genes to perform a 5-nearest-neighbour classification. This was how we assigned the class membership of each specimen to the majority of its neighbourhood that was made up of 5 specimens which had the shortest distances to it. This process was repeated for 10,000 times for each n. The corresponding averaged accuracies for the size of gene set, 2n, are plotted**.** The optimal n for best discriminating (classifying) accuracy for AS and non-SARS specimens was around 30.
