# Supplementary material for: Molecular signature of clinical severity in recovering patients with severe acute respiratory syndrome coronavirus (SARS-CoV)
Source: BMC Genomics. 2005 Sep 21;6:132. doi: 10.1186/1471-2164-6-132 (PMC1262710; doi:10.1186/1471-2164-6-132)
Supplement: Additional File 5 — Euclidean distance matrix of 55 specimens with 52 selected genes using different seriation algorithms. [file 1471-2164-6-132-S5.doc]

**Additional file 5.**

**Euclidean distance matrix of 55 specimens with 52 selected genes using different seriation algorithms.**

In order to evaluate the performance of the GAP elliptical seriation algorithm on the Euclidean distance matrix of 55 specimens with 52 selected genes, five other permutation schemes are also tested to sort the same matrix. (a) Original order of NC(11)-AS(11)-RS(33); (b) Average-Linkage-Hierarchical-Tree permutation with input order in (a); (c) One-dimensional Self-Organizing-Map order; (d) Average-Linkage-Hierarchical-Tree permutation with input order in (c); (e) GAP elliptical order; (f) Average-Linkage-Hierarchical-Tree permutation with input order in (e). Total 55 samples of AS (red ●), NC (blue █) and RS (red ○) are indicated.
