# Supplementary material for: Molecular signature of clinical severity in recovering patients with severe acute respiratory syndrome coronavirus (SARS-CoV)
Source: BMC Genomics. 2005 Sep 21;6:132. doi: 10.1186/1471-2164-6-132 (PMC1262710; doi:10.1186/1471-2164-6-132)
Supplement: Additional File 6 — Analyses of gene expression in MMP-7 and MMP-9, both of which are involved in innate immunity. [file 1471-2164-6-132-S6.doc]

**Additional file 6.**

**Analyses of gene expression in MMP-7 and MMP-9, both of which are involved in innate immunity.**

Fold changes of gene expression (y axis) in 44 SARS specimens (11 AS labeled as solid red spots; 33 RS as red circles) and 11 normal controls (blue rectangles) were plotted against the severity rank of SARS (x axis), which was derived from the generalized associated plot analysis described in **Figure 3**.

Severity rank of SARS
